# Manufacturing Parameters, Materials, and Welds Properties of Butt Friction Stir Welded Joints–Overview

**DOI:** 10.3390/ma13214940

**Published:** 2020-11-03

**Authors:** Aleksandra Laska, Marek Szkodo

**Affiliations:** Department of Materials Engineering and Bonding, Faculty of Mechanical Engineering, Gdansk University of Technology, Narutowicza 11/12, 80-233 Gdansk, Poland; mszkodo@pg.edu.pl

**Keywords:** friction stir welding, FSW, solid type welding, mechanical properties, weld strength

## Abstract

The modern and eco-friendly friction stir welding (FSW) method allows the combination of even such materials that are considered to be non-weldable. The development of FSW technology in recent years has allowed a rapid increase in the understanding of the mechanism of this process and made it possible to perform the first welding trials of modern polymeric and composite materials, the joining of which was previously a challenge. The following review work focuses on presenting the current state of the art on applying this method to particular groups of materials. The paper has been divided into subchapters focusing on the most frequently used construction materials, with particular emphasis on their properties, applications, and usage of the FSW method for these materials. Mechanisms of joint creation are discussed, and the microstructure of joints and the influence of material characteristics on the welding process are described. The biggest problems observed during FSW of these materials and potential causes of their occurrence are quoted. The influence of particular parameters on the properties of manufactured joints for each group of materials is discussed on the basis of a wide literature review.

## 1. Introduction

Friction stir welding (FSW) is a method invented at the Welding Institute of the United Kingdom and patented by Wayne Thomas in 1991 [1]. It is considered to be one of the most prospective material joining developments in the last 30 years. Primarily, this method was dedicated to joining aluminum and its alloys, but today it is widely used for titanium and its alloys, magnesium and its alloys, steel and ferrous alloys, and copper, but also polymers and composites. The FSW process is defined as a solid-state method. Materials to be joined do not melt during the process. Since the melting point is not reached, typical problems of fusion welding techniques are eliminated. These problems are usually related to a change of state, such as changes of volume and solubility of gases, and these effects are not observed during friction stir welding process [2,3,4].

**Figure 1:**
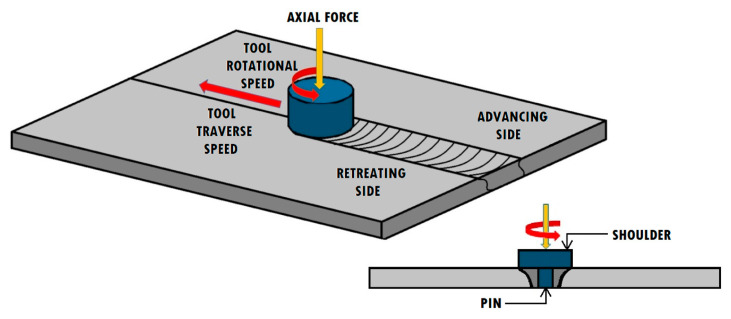
Schematic illustration of a friction stir welding process.

During the process, a specially designed tool is put into linear movement along a joint line, rotating at the same time. The kinetic energy of the tool is transformed into thermal energy, generated by the friction on the interface between the tool and the components. The heated material is plasticized by a tool and extruded around the pin in a backward direction of a tool moving along the edge of a contact line. The FSW method is usually used to produce butt welds, but it also allows the fabrication of joints of other types, such as corner welds, T-welds, lap welds, and fillet welds [5,6,7,8]. A schematic illustration of a friction stir welded butt joint is shown in Figure 1. Nowadays, the FSW method is widely used in many industrial areas, such as aerospace (wings, fuel and cryogenic tanks, fuselages) [9,10,11,12], railways (underground carriages, wagons, container bodies) [13,14,15], marine and shipbuilding (deck panels, hulls, booms, masts, offshore accommodation) [14,16], construction industry (frames, bridges, pipelines) [17,18] and land transportation (wheel rims, mobile cranes, tail lifts) [19]. The FSW technology is also applied in sectors such as machinery equipment, electronics, metalworking, and the R&D sector [20].

**Figure 2:**
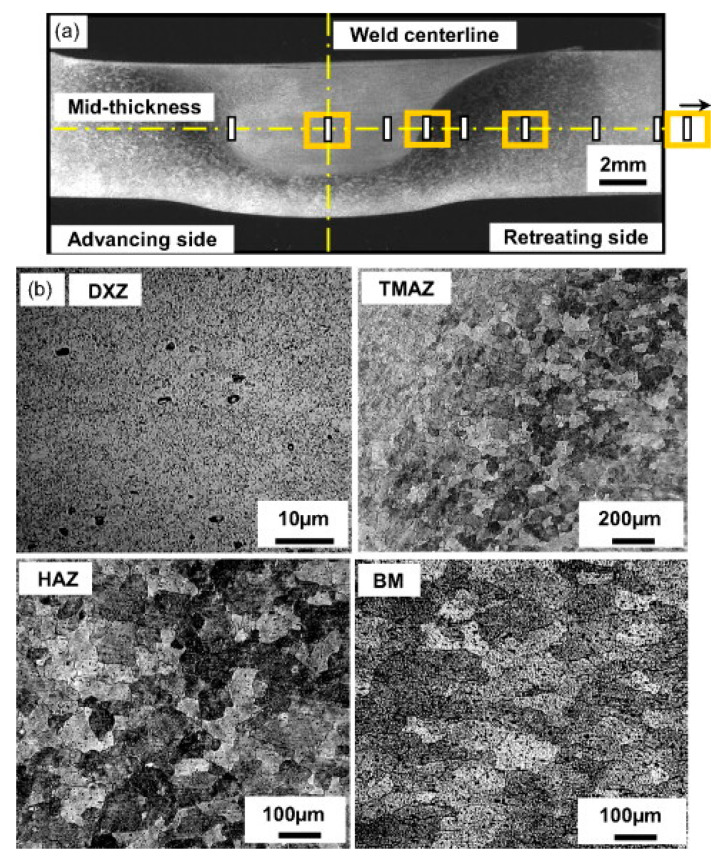
(**a**) Typical weld macrostructure of 6061–T6 Al alloy in FSW, (**b**) microstructure of the joint-stir zone (DXZ, dynamic recrystallized zone), TMAZ, HAZ, and BM taken at yellow square marks shown in (**a**) [27].

In the cross-section of the friction stir welded joint, a specific microstructure is observed. Due to the solid-state nature of the process, the zones that are not found in welds produced by conventional welding methods can be distinguished. Based on thermomechanical actions of the FSW tool, four distinct zones can be observed: weld nugget (stir zone, SZ), thermo-mechanically affected zone (TMAZ), heat affected zone (HAZ), and base material (unaffected zone, BM). The presence of the stir zone is a result of the recrystallization in the middle part of the thermo-mechanically affected zone. The nugget is formed by fine grain sized metal. The material of the SZ experiences plastic deformation due to the interactions with the tool. Rhodes et al. [21] and Liu et al. [22] claimed that in the recrystallized grains, a low density of dislocations is observed. However, other studies proved that the recrystallized grains of the SZ contain high density of sub-boundaries [23], dislocations [24] and subgrains [25]. Between the SZ and the HAZ, a unique transition zone, called the thermo-mechanically affected zone, can be observed. TMAZ is exposed to both temperature and deformations during the process. Because of insufficient deformations, strain recrystallization is not observed. Exposure to high temperatures during welding might cause the dissolution of precipitates in TMAZ. Beyond TMAZ, the heat-affected zone is observed. In that region, there are no plastic deformations, but it is still subjected to a thermal cycle. The alteration of properties in HAZ, compared to the base material, includes changes in ductility, toughness, corrosion susceptibility, and strength. The changes in grain size or chemical makeup are not observed [26]. The typical cross-section of the FSW joint and the microstructures of different zones are shown in Figure 2.

The FSW technology is classified as a green technology. As a melting point is not reached during the process, less energy is used in comparison to fusion welding techniques. Moreover, CO_2_ emission to the atmosphere can be significantly reduced [28]. It is relatively easy to control the process and setting optimal parameters allows for a subsequent reduction of necessary non-destructive testing. The pollutions generated by sprays for visual and magnetic inspection and exposure to radiation in the case of X-ray tests are reduced. Additionally, if the optimal parameters are set, post-weld heat treatment is not required [28,29]. It results in the reduction of CO_2_, energy consumption, and other pollutions emitted to the atmosphere. The FSW process uses a non-consumable tool; the use of shielding gas is not necessary.

One of the most important process parameters is the geometry of the tool. The tool consists of a specially designed pin and shoulder. The movement of the plasticized material depends on its geometrical features [30]. Its geometry also conditions the workpiece thickness, possible materials to be welded, and type of the joints [7]. The pin is an element of the tool that is directly plunged between the surfaces of the workpieces. It is plunged into the material until the shoulder reaches the contact with the surface of the components [31]. The tool design governs the process loads and microstructure of the weld, when from the heating aspects, the most important parameter is a ratio of a shoulder diameter to a pin diameter [32]. The FSW method is mostly characterized by two parameters related to its kinematics—rotational speed (ω) and traverse speed (v) along the joint line [33]. Selecting the optimum tool traverse and rotational speed is a crucial concern in the design of the FSW process.

The FSW technique allows the joining of many types of materials, even hard materials such as steel and engineering alloys. Recent studies have also been investigating the joining of metals to polymers through the FSW technique [34,35,36]. According to the analysis of Magalhaes et al. [20], most of the research and patents on FSW welding have concerned aluminum and its alloys, followed by ferrous alloys, magnesium, titanium, and their alloys. There are also studies regarding the welding of composites, copper, and polymers. The published papers analyze not only the FSW industrialization [37,38,39,40], but also the process mechanism, welding parameters, weld properties, and material microstructure. This paper presents the latest results of research on various materials welded using the FSW method and the influence of technological parameters of the process on the mechanical properties of welds and their microstructure, with particular reference to butt joints.

## 2. Methodology

The purpose of the overview is to establish the influence of the process parameters on the mechanical properties of the joints of different groups of materials. The method underpinning this paper is a systematic literature review. The number of papers published on FSW technology grows quickly, and the studies have become very diverse, making important the understanding and assessment of its impact on research and development level. In order to achieve this objective, an analysis of the literature published since 1991 to the current date was performed. The bibliographical references analysed were selected mostly from Scopus and ScienceDirect databases, using keywords such as “FSW”, “friction stir welding”, “solid state welding”, and “solid type welding”. Several hundred papers from the top journals publishing on FSW were analysed. Almost 70% of the articles presented in the review have been published in the last ten years; therefore, the paper is an analysis of the latest developments in the field of FSW process. A meticulous literature review on the applied FSW welding parameters allowed the presentation of the properties of the produced welds, if such information was presented in the quoted articles.

## 3. Aluminum and Its Alloys

Aluminum and its alloys are materials widely processed by FSW. Due to the difficulty of welding aluminum using traditional methods, FSW offers an excellent solution for joining these materials, ensuring reliability, ease of control of process parameters, and minimized risk of defects contributing to a reduction in the mechanical properties of the welds. During the FSW process of aluminum and its alloys, the temperature usually stays below 500 °C [41,42,43]. The experimental validation of the temperature on the tool surface is difficult to identify due to large deformations at the interface between the material and the tool, but Colegrove et al. [44] suggested that it can be near the solidus temperature.

Aluminum alloys can be divided into precipitation-hardened and solid solution-hardened alloys [32]. Although the precipitation-hardened aluminum alloys are easily welded by FSW, the heat-affected zone might be severely softened, essentially characterized by the dissolution or coarsening of the existent primary precipitates of the original thermal cycle [23]. It is reported that the hardness profile depends mostly on the precipitate distribution, and the grain size is of minor importance [23,45]. The most relevant to the hardness profile of the FSW joints of precipitation-hardened alloys is frictional heating during the process. The thermal hysteresis has an influence on distribution, size, and volume fraction of the strengthening precipitates [45].

The analysis of the state of the art has highlighted that there is no general dependence of mechanical properties of welds as a function of particular process parameters. In the studies of Krasnowski et al. [46], it was reported that the ultimate tensile strength (UTS) of the AA6082-T6 joints initially decreases as a function of the tool traverse speed and then increases, reaching the maximum value of the UTS for the relatively highest tool traverse speed at the constant rotational speed for three different tool geometries. Opposite results has been observed by Rao et al. [47] during the FSW of IS:65032 aluminum alloy at the tool rotational speed of 1300 rpm and triangular pin shape, but the square pin shape for the same tool rotational speed confirmed the relationship observed before by Krasnowski et al. [46]. The above examples clearly indicate that the shape of the tool plays a key role in the FSW process, and changing only this parameter may cause the opposite effect of other process parameters on the properties of welds. Considering the influence of the tool rotational speed on the UTS of FSW joints, it is worth quoting studies on AA6061 alloy by Emamian et al. [48]. In these studies, it was observed that for linear speed v = 40 mm/min, the initial increase in the tool rotational speed causes an increase in UTS, but when it reaches its maximum, the UTS decreases with the increase in the speed. In the same study, for the tool traverse speed of 100 mm/min, UTS initially decreases with the increase in the tool rotational speed, but when it reaches its minimum, UTS increases with the increase in the tool rotational speed. Rajendran et al. [49] investigated the influence of the tilt angle on the hardness of the nugget zone of AA2014-T6 FSW lap joints. The tilt angle of 2° resulted in the maximum value of the hardness. In the studies on Al 5754 alloy, conducted by Barlas et al. [50], it was reported that the tilt angle equal to 2° provides better mechanical properties of the joints compared to the zero tilt angle. The investigations of Peel at al. [51] showed that the ratio of the shoulder diameter to the pin diameter (D/d) equal to 3.6 resulted in the highest ultimate tensile strength and yield strength of the AA5083 FSW joints, while the studies of Khan et al. [52] proved that in the range from 2.6 to 3.2 of the D/d ratio, the lowest value resulted in the best UTS and elongation of the AA6063-T6 joints. The reason for the different results may be the shape of the tool. The above studies used pins with different geometry—Peel et al. [51] used a threaded pin, while a smooth cylindrical pin was used by Khan et al. [52]. What is more, it is worth noting that the alloys with different chemical compositions were used in the studies, especially in the magnesium content, which could also affect the results obtained.

In the literature, there are few reports about FSW welding of metallic foams. However, the FSW technique is not an ideal solution to join the foams due to their compressibility. The pressure necessary to create frictional forces between the tool and the material is not sufficient after inserting the tool between the components to be joined, or the resulting forces destroy the porous structure of the material. A more popular solution is to implement the FSW method to produce sandwich structures, where a porous structure is placed between two sheets of solid material. In the research of Peng et al. [53], FSW was adopted to prepare aluminum foam sandwich. For this purpose, aluminum foam and solid aluminum AA6061-T6 plates. The aluminum foam panel was inserted between two solid plates and welded on both sides. It was concluded that the FSW technology offers better mechanical properties of the foam sandwiches compared to traditional adhesion and brazing. Busic et al. [54] investigated the influence of tool traverse speed and tilt angle on the mechanical properties of FSW of aluminum foam sandwich panels. Butt welds were produced by double side welding applying insertion of extruded aluminum profile. The studies proved that both tool traverse speed and tilt angle have significant influence on the UTS and flexural strength of the welds. In general, the current state of the art is poor in this type of research. The joining of foams still needs more studies, especially when permanent metallurgical bonding has to be obtained.

It is widely reported that the process parameters play a crucial role in the mechanical properties of the welds. However, the above examples prove there are no generally defined relationships. Table 1 summarizes analyzed studies on friction stir welding of different aluminum alloys. Table 1 presents the selected parameters that provided the highest mechanical properties of the welds, and in parentheses, the properties of the parent material are given for each example.

## 4. Magnesium and Its Alloys

Among commonly used structural materials, magnesium has the lowest density. Because of its hexagonal close-packed (hcp) structure at room temperature, the formability of magnesium is very constrained; however, it increases significantly at temperatures of 230–310 °C [60]. Most of the commercially used magnesium alloys are ternary alloys containing aluminum, zinc, silicon, and rare earth metals [61]. In Mg-Al series, the most common alloys are AZ (Mg-Al-Zn) and AS (Ag-Al-Si) [62,63]. The successful method to join magnesium alloys is arc welding, but some difficulties might occur in joining, especially the cast grades alloys [60]. During fusion welding of aluminum alloys, the shielding gases are necessary due to oxidation at welding temperatures. The most significant problems occurring during fusion welding of magnesium alloys are the porosity of the welds [32], distortions due to high thermal conductivity and thermal expansion of magnesium alloys [10], evaporation, and solute atoms segregation, which leads to softening of the joint area. In addition to the application of the FSW technique mentioned above, friction stir welded magnesium alloys find their applications in industrial equipment of nuclear energy, due to their low neutron absorption, excellent thermal conductivity, and good resistance to carbon dioxide [64].

There are plenty of studies on the microstructure of the FSW magnesium joints. Xin et al. [65] reported that the primary texture does not significantly affect the final microstructure and texture of the nugget zone. However, texture distribution in the thermo-mechanically affected zone influences the mechanical properties of the joints [66]. In the studies of Yang et al. [67], it was reported that the shoulder size does not have an impact of texture modification in the nugget zone of friction stir welded Mg-3Al-1Zn alloy, but it weakens the (0002) texture in the thermo-mechanically affected zone. Commin et al. [68] observed that during FSW of AZ31 hot-rolled base material, the structure is not significantly changed when the shoulder diameter is equal to 13 mm, but the shoulder diameter of 10 mm resulted in the strong texture modification. In their studies, it was also reported that the highest tensile residual stress was observed in the thermo-mechanically affected zone. It was observed that a larger diameter of the shoulder reduced the residual stress due to the higher heat delivered to the welded material.

The analysis of the research conducted so far does not allow the drawing of general conclusions concerning the optimization of process parameters. On the basis of studies carried out by Lim et al. [69], it was concluded that the tensile properties of AZ31B-H24 welds are not significantly affected by FSW process parameters, whereas Lee et al. [70] reported that with an increase of the tool rotational speed, the strength of the joints of the same alloy increased. Wang et al. [11] and Kumar et al. [71] reported that for AZ31 butt welds, the UTS, yield strength, elongation, and hardness primarily increase with an increase of the tool traverse speed and after reaching the maximum value, decrease with a further increase of the welding speed. In the studies of Han et al. [72], the ultimate tensile strength of Mg-Gd alloy increases with an increase of the tool traverse speed. The opposite dependence was presented by Sahu et al. [73] for AM20 butt welds. It should be noted that both tests were performed at a different tool rotational speed, so the amount of heat generated was different. Moreover, both tests were different in the geometry of the tools used. In the study of Sahu et al. [73], the influence of D/d ratio on the mechanical properties was also investigated. In the range from 2 to 4, the highest D/d ratio provided the highest UTS. Sevvel et al. [74] and Pareek et al. [75] investigated the influence of the tool rotational and traverse speed on the mechanical properties on AZ31 magnesium alloy. The results of the tests do not allow the drawing of a general conclusion. Sevvel et al. [74] proposed the lowest tool traverse speed and the highest rotational speed to obtain the highest ultimate tensile strength and the highest yield strength of the welds, while in the studies of Pareek et al. [75], the highest tool traverse speed and the highest rotational speed resulted in the best mechanical properties of the welds. Sevvel et al. proposed the tool rotational speed equal to 1000 rpm, while in the studies of Pareek et al., it was set as 2000 rpm. In this case, a higher tool traverse speed could provide enough heat to the weld, which could be insufficient if the speed was lower, as in the studies of Sevvel et al. Studies on the hardness of friction stir welded magnesium alloys show contradictory conclusions. Esparza et al. [76] and Park et al. [77] reported that the welds exhibit almost the same hardness in the various zones. On the contrary, Xie et al. [78] and Zhang et al. [79] noted that the nugget of the welds has significantly higher hardness than the other zones. It can be explained by breaking up large intermetallic compounds Al_2_Ca in Mg-Al-Ca alloy studied by Zhang et al. [79] and Mg-Zn-Y phases in Mg-Zn-Y-Zr studied by Xie et al. [78] and their dispersion in the stir zone, which resulted in the increase of the hardness. As mentioned earlier, aluminum alloys are divided in two types: precipitation-hardened and solid solution-hardened alloys. Thus, in ternary magnesium alloys containing aluminum as the main alloying element, the hardness of magnesium alloy varies according to the percentage of aluminum present in the structure.

Table 2 presents the properties of FSW joints of magnesium and its alloys and the mechanical properties of the parent material if presented by the authors.

## 5. Steel and Ferrous Alloys

The FSW method was initially dedicated to aluminum and its alloys, but with the development of this technology, other materials are successfully joined. Steel and ferrous alloys are still a challenge due to their high hardness. The biggest problem when welding steel and ferrous alloys is choosing the right tool for this process. The tool material must have high resistance to frictional wear, resistance to cracking, high strength, and resistance to chemical degradation at high temperatures achieved during the process [90,91]. Finding the proper material is a major engineering challenge. There are also studies on various ceramic options [92]. Nevertheless, composite tools made of polycrystalline boron nitride/tungsten rhenium (pcBN/W-Re) are also commonly used in the FSW of steel [93].

The friction stir welding method offers a reduction of the metallurgical changes in the heat-affected zone due to lower heat input compared to fusion welding techniques. The FSW method is a good alternative for joining difficult to fusion weld steel grades. Furthermore, during fusion welding, the normal source of hydrogen might lead to hydrogen cracking, while this problem is eliminated during FSW [32]. Early studies on FSW of steels proved that the peak temperature during the process of 1000–1200 °C is much lower than that observed during conventional welding [10,94,95]. Hence, the region of the heat-affected zone with pearlitic steels, which becomes fully austenitic, is supposed to be narrower. Moreover, the size of the grains of austenite is expected to be finer than in the case of arc welding. The unfavorable transformations, such as untampered martensite, can be avoided in the FSW method [10].

The thermo-mechanical nature of the FSW process induces phase transformations controlled by the selection of appropriate process parameters, such as tool rotational speed and tool traverse speed. Changes in the microstructure of carbon steel depending on the process temperature were presented in the paper of Fujii et al. [96]. In the study of Cui et al. [97], different microstructures of high-carbon steel were observed by controlling both the tool rotational speed and the tool traverse speed. Saeid et al. [98] obtained defect-free welds of duplex stainless steel by the FSW method in relatively low temperatures, which led to the avoidance of phase transformation and the ratio between phases was not changed. In the research of Ghosh et al. [99], the dependency of temperature and rate of deformation on microstructure for high-strength M190 steel was examined. Miura et al. [100] reported that the FSW method on Cr-Mo steel results in the increase of the volume fraction of retained austenite, and the joints perform high ultimate tensile strength and elongation. A similar observation for ferritic stainless steel was noted by Fujii et al. [101]. However, there is no general explanation for this mechanism.

The influence of welding parameters of steel on the mechanical properties of welds is not fully determined. In the research of Mahoney et al. [102], the HSLA-65 alloy was welded and the mechanical properties of the welds in dependence on the rotational speed and traverse speed of the tool were examined. The results showed that the tensile strength of the welds increases with both rotational and linear speed, while the elongation of the welds decreases. The studies of Miura et al. [103] show an inverse relationship: for iron alloys with nickel and carbon, the ultimate tensile strength of the welds and their yield strength decreases with an increase in tool speed. It should be noted that these incompatibilities result from differences in the range of selected parameters. Mahoney et al. applied a tool traverse speed up to 152.4 mm/min, while Miura et al. applied one with maximum 400 mm/min. However, similarly to the studies on HSLA-65 steel alloy of Mahoney et al. [102], the elongation of the welds decreases as the rotational speed increases. In the research of Fujii et al. [104], three carbon steels with different carbon contents were subjected to the FSW method. Their tensile strength was tested depending on the established traverse speed. The results showed that for IF (interstitial-free) steels, this parameter does not significantly affect the UTS, while for S12C and S35C steels, this effect was more visible but not uniform. For S12C, UTS increased with the tool traverse speed, while for S35C, the tensile strength first increased and then decreased with an increase of the tool traverse speed. For both steels, higher UTS than the value for the parent material was achieved. The same dependence as for S35C steel was observed by Reynolds et al. [90] for DH36 steel. Tensile strength and yield strength first increased and then decreased as both traverse and rotational speed of the tool increased. The mechanical properties of the welds were higher than those of the parent material. In the research of Meshram et al. [105] on ASIS 316 steel, the highest tool rotational speed and the lowest traverse speed of the tool influenced the mechanical properties of the joints. The UTS and hardness exceeded the ones of the base metal. The studies of Maltin et al. [106] on DH36 steel showed that the highest both tool traverse and rotational speed resulted in the highest UTS and yield stress, exceeding the values for the parent material.

Some researchers claimed that FSW of steel is an attractive alternative in comparison to the fusion welding, and the feasibility of the method was proved by many studies, although more scientific research in this field is needed, especially with improving tools’ geometry and the proper selection of the tool materials [94]. In contrast, Bhadeshia and DebRoy [107] suggested that the FSW technology is not expected to be widely applied, because fusion welding techniques already allow producing reliable, cost-effective joints.

Table 3 presents the results of selected studies on friction stir welded steels. The process parameters that provided the best mechanical properties of the welds were presented, and the properties of the parent material were given in brackets.

## 6. Titanium and Its Alloys

Titanium and its alloys are characterized by very good mechanical properties such as high strength, high corrosion resistance, and a very good strength-to-weight ratio, but their processing at temperatures higher than 550 °C is difficult due to their low resistance to oxidation. In addition, in single-phase titanium alloys, a tendency to grain growth is observed, which results in a decrease in the mechanical properties of the material [115]. When using the FSW method for soft alloys such as aluminum or magnesium, the problem of tool wear and its material selection is not a challenge, but in the case of titanium joining, these problems may arise. The most commonly used tools during the FSW process are those with a developed pin geometry, such as threads, flats, or flutes. When friction stir welding titanium and its alloys, conventional tools can be significantly damaged. It is therefore necessary to modify the geometry in such a way that the material is properly mixed while minimising the problem of wear. These solutions consist of uncomplicated tools with a columnar or conical pin, and the mixing of the material is assisted by appropriate shoulder surface modification, such as scrolls, ridges, knurling, grooves, or concentric circles. Usually, the ratio of shoulder diameter (D) to pin diameter (d) is chosen to be equal to 3 to provide the best mechanical properties of the joint [116,117]. In the case of FSW of titanium and its alloys, a heat generated by the shoulder cannot flow to the join root, and a relatively small pin is not able to properly stir the plasticized material. Therefore, usually tools with smaller shoulder diameter and larger pin are used, and the D/d ratio is smaller, which can be observed in the examples summarized in Table 4. Another important aspect of the tool design process is the selection of the right material. The significant strength of titanium and its alloys at hot working temperatures makes it necessary to select a material for the tool that will be resistant to high forces during the process and be inert for reactive titanium at temperatures reaching 0.8 of its melting point. The most popular materials for FSW of titanium and its alloys are W-, Re-, Mo-based alloys, and TiC [118,119,120,121,122,123]. Titanium and its alloys have a relatively low thermal conductivity and a high melting point, so a temperature gradient between the advancing and retreating side of the components may appear when friction stir welding. Applying the FSW method to titanium alloys might be challenging due to the thickness of the components and the tool geometry limitation, mostly for alpha and near-alpha alloys. In case of such alloys, the lower thermal conductivity of alpha phase, its higher low stress, and higher heat capacity of titanium makes it difficult to select the proper tool material for titanium alloy with high β trans temperature. β or α + β alloys are susceptible to the temperature of β transus during friction welding in dependence on welding parameters and thermal distribution during the process. Examples of tools used during the process of FSW of titanium and its alloys are tungsten carbide (WC) and titanium carbide (TiC) tools produced by sintering. Specially designed water cooling systems are also successfully used to better dissipate heat from the tool [124].

The mechanical properties of welds are directly influenced by the evolved micro- and macrostructure of the joints. The macrostructure observed in titanium alloys is clearly different from the banded elliptical macrostructure observed in aluminum and its alloys [32] and a parabolic shape of the weld nugget was observed in the research of Gangwar et al. [125] on titanium alloys. Fonda et al. [126], in their research on aluminum alloys, observed that the banding may be attributed to the fluctuations in the second phase particles density or the crystallographic texture changes, while in the titanium alloys, the absence of hard second phases or inclusions suggests the formation of banding formation in the nugget due to texture difference [127]. Gangwar et al. [127] in the review suggested that the elongation of the FSW titanium components is lower than the base metal due to microstructural gradients observed in the gauge length of the transverse tensile specimen, and the strains are mostly carried by the narrow areas of the thermo-mechanically affected zone. However, the examples presented in Table 4 show the opposite conclusions. In the studies of Kulkarni et al. [128] on Ti-54M plates, the increase in specimen elongation was observed with the increase in the tool traverse speed in the FSW process. On the contrary, in the studies of the same authors on Ti-6242 plates of the same thickness and the same process parameters, the decrease of the elongation with the increase of the tool traverse speed was observed. This confirms the assumption that the chemical composition of the material is very important for FSW welding. These studies also confirm the suggestion that α and near α alloys, among them Ti-6242, are more challenging. Similarly, the studies of Su et al. [129] on Ti-6Al-4V a decrease in elongation with an increase in the tool traverse speed was observed, while Mashinini et al. [130] studies on the same alloy showed the opposite relation. This effect is caused by differences in other applied parameters, including tool geometry, tool rotational speed, and tilt angle. In the same studies of Su et al. [129] and Mashinini et al. [130], opposite correlations between UTS and the tool traverse speed were also presented. In the studies of Fujii et al. [131] on pure titanium plates, the UTS firstly increases with the increase of the tool traverse speed and then decreases with the further increase of the tool traverse speed. The opposite relation was observed by Kulkarni et al. [128] in the studies on Ti-6Al-4V titanium alloy. Kulkarni et al. [128] presented a tendency of increasing UTS as a function of the tool rotational speed, while Zhang et al. [120] reported an opposite relation. An in-depth review of the literature did not allow the explanation of the reason for contradictory conclusions in the cited examples, but it should be noted that in all cited studies, tools with different geometry and made of different material were used, and this could have been the reason for obtaining different results.

Table 4 presents the results of friction stir welding on titanium and its alloys and the mechanical properties of the parent material, if presented by the authors.

**Table 4:** FSW of titanium and its alloys—process parameters and mechanical properties of the joints.

Material	Plate Thickness [mm]	Process Parameters					Weld Properties					Reference
		v [mm/min]	ω [rpm]	Tool Shape	Pin D/d Ratio	Tilt Angle [°]	UTS [MPa]	Yield Strength [MPa]	Hardness of the Stir Zone [HV]	Elongation [%]	Defects	
Pure Ti	2	200	200	-	2.5	-	~430 (420)	-	180 (146)	-	-	[131]
Pure Ti	5.6	50	110	-	-	-	430 (440)	-	-	20 (25)	Defect free	[119]
Ti-54M fine grain	0.1	100	275	-	-	-	~950 (972)	~780 (889)	-	~5.9 (16.5)	Defect free	[128]
Ti-6Al-4V fine grain	0.1	125	325	-	-	-	~950	~760	-	~4.3	Defect free	[128]
Ti-6Al-4V standard grain	0.1	100	275	-	-	-	~930 (950)	~720 (880)	-	~7.7 (14)	Defect free	[128]
Ti-6Al-4V	2	101.6	900	Smooth cylindrical pin	1.6	2.5	1156.2 (1014.7)	1067.4(941.8)	-	21.7 (23.1)	Processing defects	[129]
Ti-6Al-4V	2	50	250	Tapered pin	2	-	813 (1013)	-	-	3.2 (8.5)	-	[128]
Ti-6Al-4V	3	75	300	Small shoulder and large tapered pin	-	-	1025.0 (higher than BM)	973.6 (higher than BM)	-	9.7	-	[132]
Ti-6Al-4V	3	60	300	Convex shoulder and tapered pin	~3	-	~1050 (~920)	~950 (~830)	~315	~33 (~21)	Cavity defects	[120]
Ti-6Al-4V	3	45	550	Tapered pin	1.75	0.5	1059 (1000)	-	-	4 (18)	Small root flaws	[133]
Ti-6Al-4V	3.17	40	500	Flat shoulder surface and tapered smooth pin	2	1.5	1040 (1017)	-	-	9 (20)	-	[130]
Ti-6Al-4V	6	100	280	-	-	-	1016 (1045)	971 (978)	335.6 (315.4)	9 (16)	-	[134]
Ti-6242 fine grain	0.1	125	325	-	-	-	~950	~730	-	~4.1	Defect free	[128]
Ti-6242 standard grain	0.1	125	325	-	-	-	~880 (1000)	~730 (895)	-	~8.4 (12)	Defect free	[128]
TC4	2	50	400	Smooth tapered pin	-	2.5	953 (1036)	-	~345 (~325)	-	-	[135]

## 7. Copper and Its Alloys

Copper and its alloys are widely used in many engineering applications due to their properties such as good electrical and thermal conductivity, relatively good mechanical strength, high corrosion resistance, and high formability. The most popular copper alloying elements are zinc, aluminum, nickel, and tin [136]. After steel and ferrous alloys and aluminum and its alloys, copper and its alloys are the most commonly used materials in industries, especially in the marine, aerospace, electronics, and military sectors. However, pure copper strength is not high enough for load bearing components, but it increases by alloying. The most popular copper alloys are solid solution hardened (single-phase). Copper is characterised by low galvanic reactivity, so the risk of reaction or corrosion is low. It is also characterized by high plasticity and resistance to oxidation. The most important applications for copper and its alloys include heat sinks, electrodes for resistance welding, and rotating target neutron sources. The FSW method was also successfully used for joining components for nuclear waste canisters [137,138]. It is also used in processes of soldering and brazing [139].

Joining copper and its alloys using traditional welding methods is difficult due to the high thermal conductivity and high melting point, which makes it necessary to generate a large amount of heat, thus increasing the cost of the process, and the resulting welds may exhibit porosity, distortion, and solidification cracks. Conventional fusion welding techniques require very fast heat delivery due to 10–100 times higher heat conductivity than that of steels [10,32]. Copper and its alloys are ranked as hard-to-weld materials [140]. The most significant problems occurring during the conventional welding of Cu and its alloys are high distortion, irregularities of the weld surface, decrease of strength at the weld surface connected with the formation of ZnO (for high Zn-content alloys), insufficient penetration because of the high thermal conductivity of Cu, and colour change because of the oxidation process. The FSW method is an excellent solution, because the melting temperature is not reached during the process.

Due to the high thermal conductivity of copper and its alloys, the required heat input should be higher than for FSW of other materials. This means that the process is usually conducted at lower tool traverse speed and/or higher tool rotational speed. It is required mostly for FSW of pure copper, which has higher thermal conductivity than its alloys. As for all friction stir welded metal joints, four specific zones can be observed in the cross-section–stir zone (SZ), thermo-mechanically affected zone (TMAZ), heat-affected zone (HAZ), and base metal (BM). For the FSW joints of copper alloys, the HAZ is not highly distinguishable [141,142]. The recrystallization process occurs relatively easily in copper and its alloys, especially single-phase, so the SZ extends almost the TMAZ and the boundaries between those two zones are hard to determine.

In the research of Machniewicz et al. [143], 5 mm pure copper plates were friction stir welded in both the longitudinal direction and perpendicular to the rolling direction. For both examples, the ultimate tensile strength decreased with an increase in the tool traverse speed. Moreover, the microhardness of the welds was measured. The microhardness profile presented a “W” shape, which is characteristic for most friction stir welded joints [144,145,146,147,148,149]. In the profile of such welds of any metal, a sharp decrease in hardness can be observed in the heat-affected zone, and then the hardness slightly increases in the direction of the weld nugget. This phenomenon is related to the difference in grain size in various zones—in the weld zone, the microstructure is more fine-grained than in the heat-affected zone, and therefore higher hardness is observed there. In the studies of Khodavardizadeh et al. [146], Xue et al. [145], and Surekha et al. [150] on pure copper plates, the ultimate tensile strength of the welds as a function of the tool traverse speed was also determined. The results of those studies are not consistent with those of Machniewicz et al. [143], and the UTS of the welds increased with an increase of the tool traverse speed. In the studies of Khodavardizadeh et al. [146] and Surekha et al. [150], it was observed that the elongation of welded samples increases with an increase of the tool traverse speed, while Xue et al. [145] noted the opposite relationship for the same material. The above quoted studies were carried out with different process parameters and the values of applied tool rotational speed were more than twice as high for tests of Xue et al. [145] as for Surekha et al. [150]. In the studies of Liu et al. [151] on pure copper plates, the ultimate tensile strength as a function of the tool rotational speed was measured. It was observed that the UTS firstly increased and then slowly decreased with the increase of the tool rotational speed. The same relation was observed while measuring the elongation. The maximum UTS was equal to the value for the base material. In the studies of Xue et al. [145] and Khodavardizadeh et al. [147], the UTS of the welds decreases with an increase of the tool rotational speed, while in the studies of Sahlot et al. [152], Xie et al. [153], and Cartigueyen et al. [154] on the pure copper plates, the opposite relationship was observed. Cartigueyen et al. [154], Xie et al. [153], and Xue et al. [145] reported that the elongation of the copper FSW joints increased with the increase of the tool rotational speed, while the opposite relationship was observed by Khodavardizadeh et al. [147]. In this study, it was also noted that the hardness of the copper weld nuggets increases with the increase of the tool traverse speed, while Surekha et al. [150] observed almost no changes of the hardness, and the measured values were similar to the one of the base material. Khodavardizadeh et al. [146] noted that the hardness of the copper weld nuggets decreases with an increase of the tool rotational speed, while Xie et al. [153] and Cartgueyen et al. [154] noted an opposite relationship. In another study of Cartigueyen et al. [155] on 6 mm thick copper plates, the influence of the pin geometry was investigated. The threaded cylindrical pin provided better mechanical properties of the friction stir welded joints than square, triflute, and hexagonal pins. Different tool geometries were used in all the quoted research or these geometries are not presented in the papers. It should be noted that tool geometry is a key process factor, and it is necessary to define it in published studies.

Table 5 shows an overview of the studies on copper and its alloys conducted so far. Each example contains a set of parameters (if given) that provided the best mechanical properties of the welds and the values of these properties including the properties of the parent material (values in brackets).

## 8. Polymers

Polymeric materials are generally characterised by different properties than metallic materials. Although the FSW method was initially dedicated to metal bonding, with the increasing use of polymers in various industries, it has been successfully used to join also this group of materials. The research on the friction stir welding of polymeric materials conducted so far focuses mainly on joining of polyethylene (PE) [164,165,166,167,168,169], high-density polyethylene (HDPE) [166,170,171,172], polyamide Nylon 6 [173,174,175], acrylonitrile butadiene styrene (ABS) [176,177,178,179], polyvinyl chloride (PVC) [171], polypropylene (PP) [180,181], and polyethylene terephthalate glycol (PETG) [182].

Using conventional tools for friction stir welding of polymers is the easiest but not the most effective approach, which will be concluded later. Panneerselvam et al. [183] successfully butt-joined 10 mm polypropylene plates using the FSW method, but for some pin geometries, the insufficient material soften; the tool damage or the blowhole defects were observed in the joint line. Design of experiments analysis (DOE) was used for modelling and analysing the influence of the process parameters [184]. The analysis of the obtained results allowed the authors to conclude that insufficient heat on the retreating side did not allow for proper mixing of the material in this zone and increased the probability of weld defects. The same conclusions were presented by Simoes et al. [185]. The morphology of the polymethyl methacrylate joints welded by the FSW method was analysed, and it was claimed that the advancing side of the welds performs almost the same transparency as the base material, while in the retreating side, the insufficient stirring and voids were observed. The studies conducted so far allow the conclusion that low heat conductivity of polymeric materials is not favourable for an efficient FSW process. Sufficient softening and plasticization of the material that is not in direct contact with the tool is difficult to achieve. In the literature, approaches can be found related to the application of an additional heating system that would compensate for heat deficiencies related to low thermal conductivity and friction coefficient of polymer materials. Squeo et al. [186] friction stir welded 3 mm polyethylene sheets using a pin previously heated with a hot air gun. This solution was not considered reliable due to the rapid cooling of the tool. Another approach was to use a hot plate between the CNC table and the components. The plate was heated up to 150 °C and ensured high quality of joints, while the biggest disadvantage of this method was low repeatability during the process. Arbegast [187] proposed a model of the FSW joint that describes the conditions of the process and a mechanism of creating weld defects. The theory also confirms that volumetric defects are likely to be observed on the retreating side of the weld.

The FSW method using a conventional tool to perform the welding process usually does not bring the expected results, and the properties of the welds are relatively low. In order to minimize the risk of weld defects and increase the efficiency of the process, a modification of the FSW method—stationary shoulder friction stir welding (SSFSW)—is used. The mechanism of the SSFSW process consists of a rotating pin that runs in a non-rotating shoulder element sliding on the material surface during welding. The stationary shoulder, usually called a shoe, minimizes the risk of the plasticized weld material being expelled from the weld seam [181]. The literature review on the SSFSW method can lead to the conclusion that this modification should be used for joining polymers in order to obtain non-defects and high mechanical properties welds [168,176,182,188]. Rezgui et al. [188] applied the SSFSW method with a wooden stationary shoulder to weld 15 mm thick HDPE. The temperature detected during the process was in a range from 120 to 180 °C, which means that the material reached its melting point, and the process did not have a solid-state nature. Other studies also confirm that friction stir welding of polymers is not a solid-state process, unlike the FSW of metals [185,189]. In order to obtain better results from the SSFSW method, a tool called “hot shoe” was developed and patented by Nelson et al. [190]. The aluminum static shoulder with the polytetrafluoroethylene (PTFE) coating with a heater component inside of the shoe allows the attainment of the tensile strength of 75% of the base material of ABS. The concept of SSFSW with a hot shoe as a shoulder was also successfully used in the studies of Bagheri et al. [176], Banjare et al. [191], and Laieghi et al. [192].

Sahu et al. [180] successfully welded 6 mm polypropylene sheets using three pin geometries—cylindrical, square, and conical. Only cylindrical and square pins enabled the production of defect-free welds. The influence of the tool traverse speed and the tool rotational speed on the ultimate tensile strength was determined. It was observed that the UTS of the welds firstly increased with an increase in the tool rotational speed and then slowly decreased. The same dependence was observed for the tensile strength function of welds on the tool traverse speed. The same conclusions for both tool traverse and tool rotational speed were confirmed by Pirizadeh et al. [178] on the friction stir welded 5 mm thick ABS plates and Arici et al. [165] on double pass friction stir welded Nylon 6 plates, where the influence of the tool traverse speed on the UTS of the welds was measured. In the studies, it was concluded that the tilt angle of the tool equal to 1° results in better mechanical properties of the welds than the tilt angle of 0°. The studies of Youssif et al. [175] on 13 mm thick Nylon 6 plates proved that the UTS of the welds decreases with an increase of the tool traverse speed. The same dependence was observed for the UTS as a function of the tool rotational speed. The same conclusions were presented in the studies of Zafar et al. [174] on 16 mm Nylon 6 plates. In the studies of Bagheri et al. [176] on 5 mm thick ABS plates, the UTS always increased with the increase of the tool rotational speed for all of the values of applied tool traverse speed. The maximum value of UTS reached almost 90% of the value for the base material.

Table 6 summarizes the best process parameters applied during the friction stir welding of polymeric materials and presents the mechanical properties of welds and parent material if given by the authors.

## 9. Composites

Polymer matrix composites (PMCs) and metal matrix composites (MMCs), especially aluminum matrix composites (AMCs), replace metals and their alloys and polymers in many industries, as the mechanical properties of such materials can be controlled by the proper selections of the filler properties. High temperature during conventional welding methods of metal matrix composites can lead to degradation of the microstructure of the composite, which leads to deterioration of mechanical properties of the joint. As an example, the process of formation of a brittle Al_4_C_3_ phase during conventional welding of a SiC-reinforced aluminum matrix composite can be presented [60]. Phase changes might be avoided by using shorter thermal cycles or lower heat input. During the FSW process, the temperature of the components to be joined is also increased, but it is relatively lower than that during conventional welding processes, and this problem is reduced. The problems that might occur during the FSW of composites include, beyond in the case of MMCs, the possibility of forming the brittle phases, which results in deterioration of strength, the formation of clusters of reinforcement particles, or change of particles’ configuration due to the plastic deformation and applied forces and temperature [194,195].

In the FSW process of composites, in addition to the standard process parameters such as the rotational and traverse speed of the tool and the tilt angle of the tool, the shape of the tool itself also has a significant impact on the properties of the joints. Vijay and Murugan [196] used three pin shapes to friction stir weld Al/TiB_2_/10 composite—square, hexagonal, and octagon. Using the untampered square pin resulted in obtaining the maximum tensile strength of the joint equal to 99.47% of the base material. The authors explained that by obtaining the highest ratio of static volume to dynamic volume of the plasticized material equal to 1.56 for the square pin, the best mechanical properties of the joints were achieved, while for hexagonal and octagon pins, the values were equal to 1.21 and 1.11, respectively. The same phenomenon was confirmed by Hassan et al. [197] in the study on aluminum matrix composite containing Mg, SiC, and graphite particles. The square head pin provided better mechanical properties of the joints than hexagonal and octagonal pins. Mahmoud et al. [198] used four different pin shapes—circular with and without threads, triangular, and square—to fabricate composite surface layers with SiC particles dispersed in A1050-H24 aluminum plates. It was reported that the square pin enabled the formation of the most homogeneous microstructure of the nugget zone.

Another aspect often discussed in the case of FSW of composites is the change of shape and size of the particle size of the composite reinforcement. The first reports in this area indicated the identical number of particles before and after the process, which implies that there is no particle breakage during the process [199,200,201]. However, other tests conducted prove that the breakdown of the reinforcing particles takes place in the nugget zone during the FSW process [202,203,204,205,206]. Baxter and Reynolds [202] reported that for the composite with 7079 aluminum matrix and SiC reinforcement, the number of SiC particles the number of particles doubled without changing their volume percentage, which means that the particle breakage takes place during the process. In the studies of Acharya et al. [207], the particle size of the SiC reinforcement in AA6092 in friction stir welded material zones was measured. In the nugget zone, thermo-mechanically affected zone, and the base materials the size of the particles was equal to 4.03, 4.99, and 7.92 µm, respectively. Feng et al. [204,205], in the studies on Al2009-15vol% SiC composite, reported that the particle breakage takes place in the stir zone, and the particles are uniformly distributed. Moreover, it is generally observed that the matrix phase experienced a grain refinement due to the dynamic recrystallization resulting from the frictional heating [204,205,206].

Kumar et al. [208], in the studies on glass-filled Nylon 6 friction stir welded 5 mm thick plates, used different tool traverse and tool rotational speed, and the values of the tilt angle were equal to 0, 1, and 2°. It was observed that the highest ultimate tensile strength and the elongation were obtained for the joints with the highest tilt angle. The UTS of the joints increased with an increase of the tool rotational speed and decreased as a function of the tool traverse speed. In the studies of Bhushan et al. [209] on the AA6082/SiC/10p 6 mm thick plates, the lowest tilt angle of 1° and the highest tool rotational speed resulted in the highest UTS and elongation. Jafrey et al. [210] reported that for 5 mm thick plates of PP/C30B/EA nanocomposite, the highest tool traverse speed resulted in the highest UTS of the joints. Liu et al. [211] and Wang et al. [212], in their studies on AC4A/SiC/30p and 2009Al-T4/SiC/17p, respectively, also reported that the elongation and the UTS of the joints increase when the tool traverse speed increases. Mozammil et al. [213], in the studies on Al-4.5% Cu/TiB_2_/2.5 p plates with thickness of 6 mm, noted that the highest tool traverse speed of 26 mm/min and the highest tool rotational speed of 931 rpm resulted in the highest UTS for all the joints fabricated with three tool shoulder geometries—full flat, 1 mm flat shoulder and 7° concave, and 2 mm flat and 7° concave. Among them, the tool with 1 mm flat shoulder and 7° concave provided the best mechanical properties of the joints. Vijayavel et al. [117] investigated the influence of the D/d ratio on the mechanical properties of the FSW welds of LM25AA-5% SiC. Among the values from 2 to 4 in steps of 0.5, the D/d ratio equal to 3 resulted in the highest UTS, hardness, and elongation of the welds. The UTS value of such a joint reached 123% of that for the base material.

Table 7 summarizes the process parameters and weld properties for composites, considering in brackets the properties of the parent material if given by the authors.

## 10. Dissimilar Materials

Joining of dissimilar materials is essential in applications that require different material properties in the same component, i.e., joining different aluminum alloys to other metals allows reducing weight [223]. However, FSW of different metals is very challenging due to the huge differences in mechanical and metallurgical properties of dissimilar materials [224,225]. Welding of dissimilar materials in the context of FSW may also refer to joining the same material family but different alloys or grades, or the same material but with different thickness of components. Recently, the FSW method has been successfully and progressively applied to weld dissimilar alloys because of its technical benefits and cost-effectiveness. Comprehensive analysis of material flow during the FSW process of dissimilar materials, studies on mechanical properties of joints, and selection of process parameters are necessary before applying the method in constructional applications [226,227]. One of the key parameters, besides the geometry of the tool and its rotational and traverse speed, is the offset of the tool toward one side. It should be noted that it is difficult to predict the amount of heat generated, material flow, and mechanical properties of dissimilar joints using theoretical analysis [228].

The FSW method allows for avoiding problems that occur during fusion welding. For instance, arc welding, laser beams, and electron beams might cause the formation of coarse grains or brittle intermetallic compounds in the dissimilar aluminum/magnesium joints [229,230]. The proper selection of welding parameters using the FSW method is extremely difficult in the case of aluminum–magnesium welding due to the possibility of liquid formation resulting from the relatively low eutectic temperature (437 and 450 °C) in the binary Al-Mg phase diagram [231]. This phenomenon may, as in the case of fusion welding techniques, result in the formation of intermetallic compounds. Abdollahzadeh et al. [232] improved the microstructural characteristics by using a zinc interlayer in the magnesium and aluminum butt joints. Intermetallic formation of Al-Mg was avoided. The most common phases in the stirred zone were both Mg-Zn and Mg-Al-Zn intermetallic compounds, as well as Al solid solution and residual Zn. It is also difficult to obtain aluminum to copper welds using FSW. The brittle intermetallic compounds such as AlCu, Al_2_Cu, and Al_9_Cu_4_ are easy to create in such joints [224,233,234]. Zhang et al. [235] proposed the underwater process environment to minimize this problem.

Movement of the material around the pin during FSW of dissimilar materials affects the creation of the bond formation and material interlocking mechanism. It improves the mechanical properties of the weld and increases its strength. This phenomenon was reported for Al-Mg [236], Al-Cu [237], Mg-steel, and Al-steel [238] welds. The phenomenon of material interlocking depends on the characteristic of the complex material flow and depends mostly on the geometry of tool and the positioning of components to be welded. The mechanical interlocking occurs only in a stir zone, and it is an effect of the rotational movement of the tool. The phenomenon of interlocking is also observed in explosive welding where a periodic structure can be distinguished, but this organised structure is not observed in the case of friction stir welding [239].

As established by Fu et al. [240], the heat generated during the FSW process mostly derives from the friction (E_f_), the viscous dissipation (E_v_), and the plastic deformations at the interface between tool and components (E_d_). Zettler et al. [241], in the studies on dissimilar FSW of AZ31 and AA6040 alloys, reported that at the boundary between magnesium alloy and the tool, the frictional coefficient is lower than that on the aluminum alloy and the tool boundary. It was recommended to give tool offset toward aluminum to provide greater contact between the tool and the aluminum to increase the contributions of E_f_ and E_v_ during the friction stir welding process. This phenomenon can be explained by referring to the crystal structures of these materials. In the case of aluminum with its face-centred cubic structure (FCC) and twelve slip systems, better deformability is observed in comparison to the hexagonal close-packed structure (HCP) with three slip systems of magnesium. It promotes higher heat input through E_v_ and E_d_ during the FSW of aluminum than of magnesium [223,240]. These types of phenomena have a significant impact during the FSW of dissimilar materials; therefore, an important process parameter in such cases is also the tool offset.

In the studies of Jamshidi et al. [31] two aluminum alloys were used to fabricate friction stir welded dissimilar joints—AA6061-T6 and AA5086-O. For the case with AA6061-T6 plate on the advancing side and AA5086-O plate on the retreating side, better UTS and yield strength of the join were observed, but the values of the reverse configuration were only slightly worse. Moreover, for both cases, it was observed that the highest tool traverse speed of 150 mm/min and the lowest tool rotational speed of 840 rpm resulted in the best mechanical properties of the joints. Kasai et al. [242] fabricated three dissimilar material joints—on the advancing side, low carbon steel plates were used, while on the retreating side, pure magnesium, AZ31, and AZ61 plates, respectively, were used. The highest UTS was noted for the joint with low carbon and AZ61, and it was observed that the UTS of steel/magnesium joints increases with the increase of Al content in the magnesium alloy. The reason for this phenomenon is simple—for all the used magnesium components, the UTS of the base material also increases with the increase in aluminum content in the material. In the studies of Zettler et al. [241], the dissimilar joints of Al6040-T61 and AZ31 were fabricated using the FSW method. The UTS of the joint of the configuration with aluminum alloy on the retreating side was about 50% higher than for the joint with reverse configuration. Peel et al. [243] used AA5083 and AA6082 aluminum alloys to fabricate dissimilar joints. It was observed that for both material configurations, the highest UTS was achieved when the tool traverse speed and the tool rotational speed were the highest and equal to 300 mm/min and 840 rpm, respectively. Furthermore, the joints with AA5083 plate on the advancing side exhibit higher UTS than for the reverse configuration, and for all the joints, no defects were observed. Similar studies were conducted by Khodir et al. [244]. The dissimilar welds of AA2024-T3 and AA7075-T6 were fabricated with both plates configurations. The welds with AA2024-T3 plate on the advancing side exhibit better mechanical properties, such as the UTS, the yield strength, and the elongation. Avinash et al. [245], in studies on AA2024-T3 on the advancing side and AA7075-T6 on the retreating side joints, reported that applying the lowest tool traverse speed of 80 mm/min and the highest tool rotational speed of 1400 rpm resulted in the highest UTS of the joint. Palanivel et al. [246] investigated the influence of the tool geometry on the UTS of dissimilar AA6351-T6 on the advancing side and AA5083-H111 on retreating side joints. Five different pins were used: Straight square, straight octagon, straight hexagon, tapered octagon, and tapered square. Among them, the straight square pin enabled the production of defect-free joints with the highest UTS, while all the welds fabricated with tapered octagon and tapered square pins exhibit tunnel defects along the joint line. Malarvizhi et al. [247] studied the influence of the D/d ratio of the tool on dissimilar AZ31B-O/AA6061-T6 joints. For the tapered smooth pin, the D/d ratios from 2 to 4 in steps of 0.5 were investigated. It was reported that the value equal to 3.5 resulted in the highest UTS, yield strength, and elongation of the produced joints.

Table 8 summarizes the studied results of research on welds from dissimilar materials. Table 8 includes the materials used for advancing and retreating side of the welds, process parameters, welds properties, and the properties of base materials if given by the authors.

## 11. Conclusions and Future Challenges

Over the past two decades, FSW technology has developed significantly, and it is widely used to combine not only aluminum and other light metals but also titanium, steel, composites, and polymers. The above literature review has extensively discussed the current state of knowledge and addressed the most common problems arising in the process of welding different groups of materials. The analysis of material characteristics and the correct selection of parameters for a particular material is crucial for effective welding.

Despite numerous studies on the selection of suitable process parameters such as tool traverse speed and tool rotational speed, tool geometry, tilt angle, and tool offset, there is still a need for process optimization. It should not be forgotten that in addition to the main process parameters, consideration should be given to factors such as plunged depth, axial force, and tool material. In addition, the material flow mechanism needs to be standardized, especially when welding dissimilar materials with different mechanical properties. Optimization of process parameters should be based on the characteristics of the materials being processed. Appropriate tool geometry and its material selection should also be preceded by a material analysis. In the case of aluminum or copper welding, the problem of material wear is not as important as in the case of titanium or steel. For these materials, specially designed materials are used, most often composite materials, which allow minimizing the process of material wear. Titanium welding requires a tool with an uncomplicated geometry, while for light alloys, a more developed tool geometry is required. SSFSW technology is becoming increasingly important for polymer welding, while traditional FSW technique might be problematic with a conventional tool, it usually does not bring the expected results, and the properties of the welds are relatively low. Despite extensive knowledge of the welding of aluminum and its alloys, the problem of the welding of dissimilar materials still remains. The most recent reports indicate the use of an interlayer or water environment to minimize the risk of unwanted intermetallic compounds.

The environmental friendliness and cost-effectiveness of this method make its use increasingly widespread in many industries, but further work is needed to optimise this process to unify the conclusions about the impact of individual parameters on the properties of the resulting welds. It is also necessary to work on further improving the geometry of the tool and the proper selection of its material in order to minimize the wear process. For the development of friction stir welding technique, it is important to control tool wear state in the real time of the process in order to ensure the highest possible process repeatability. The tool life can be effectively predicted by implementing the numerical simulation of the interaction between the component’s material and the tool. The material flow is the crucial phenomenon during FSW, and it still requires more understanding. There is a need for concerted research efforts towards the computer simulations of the process, which will help develop understanding of the mechanism of material flow and heat generation. Due to the wide application of FSW technology in the marine industry, special attention should be paid to the electrochemical properties of the produced welds. The current state of the art is poor in this type of research. Future studies should focus on the influence of particular process parameters not only on the mechanical properties of welds intended for the marine industry, but also on their corrosion properties.

## Figures and Tables

**Table 1:** FSW of aluminum and its alloys—process parameters and mechanical properties of the joints.

Material	Plate Thickness [mm]	Process Parameters					Weld Properties					Reference
		v [mm/min]	ω [rpm]	Tool Shape	Pin D/d Ratio	Tilt Angle [°]	UTS [MPa]	Yield Strength [MPa]	Hardness of the Stir Zone [HV]	Elongation [%]	Defects	
AA2195-T8	7.4	300	400	Cone shape threaded pin, threaded surface of the shoulder	-	-	445.0 (607.9)	-	-	12.50 (12.49)	No defects	[55]
AA356.0-T6 (double side welded)	8	200	1200	Threaded conical pin	3.25	-	200 (244)	123 (140)	-	16.3 (13.4)	-	[56]
AA5083	3	100	-	M6 threaded pin	3.6	2	304 (457)	154 (392)	-	-	-	[51]
AA5086-O	5	150	900	Tapered pin with 3 threads and concave shoulder surface	3.3	-	250 (253)	123 (112)	-	-	-	[31]
AA6013-T4	2.5	450	1400	-	-	-	300 (320)	-	-	-	-	[25]
AA6013-T6	2.5	400	1400	-	-	-	295 (394)	-	-	-	-	[25]
AA6061	5	100	1300	Cylindrical smooth	-	-	227 (308.5)	~153 (266.6)	-	~7.3 (16.28)	Defect free	[47]
AA6061	10	100	1600	Threaded cylindrical pin	3	-	214.4 (305)	-	-	-	No defects	[48]
AA6061-T6	5	150	900	Tapered pin with 3 threads and concave shoulder surface	3.3	-	285 (315)	241 (278)	57	-	-	[31]
AA6061-T651 (double side welded)	8	200	1200	Threaded conical pin	3.25	-	218 (299)	142 (264)	-	23.3 (27.2)	-	[56]
AA6063	5	100	2800	Threaded cylindrical pin	3	3	~150 (220)	~70 (170)	-	4.5 (13)	-	[57]
AA6063-T5	4	600	-	-	-	-	~155 (~220)	~105 (~185)	-	10 (~19)	No defects	[58]
AA6063-T6	4.75	40	900	Cylindrical smooth pin	2.6	1.5	145.34 (220)	-	-	20.85 (14.00)	-	[52]
AA6082-T6	8	900	710	Cylindrical pin with threads and three flutes	3.3	1.5	243.4	-	-	-	-	[46]
AA6352 (double side welded)	6	115	1350	Tapered pin	-	2	172 (250)	-	90.2 (93.5)	-	No significant defects	[33]
AA7050-T7451	6.35	103	396	-	-	-	429 (555)	304 (489)	-	6 (16.7)	-	[24]
AA7075-T651	6.35	127	-	-	-	-	525 (622)	365 (571)	-	15 (14.5)	-	[42]
SSM 356	4	160	1750	Cylindrical pin	4	3	173.5 (168.7)	138.8 (134.9)	40.9 (36.4)	3.1 (5.3)	-	[59]
SSM 356-T6	4	160	1750	Cylindrical pin	4	3	172.9 (295.6)	138.3 (236.5)	68.4 (61.2)	4.5 (4.8)	-	[59]

**Table 2:** FSW of magnesium and its alloys–process parameters and mechanical properties of the joints.

Material	Plate Thickness [mm]	Process Parameters					Weld Properties					Reference
		v [mm/min]	ω [rpm]	Tool Shape	Pin D/d Ratio	Tilt Angle [°]	UTS [MPa]	Yield Strength [MPa]	Hardness of the Stir Zone [HV]	Elongation [%]	Defects	
AM20	4	63	600	Cylindrical pin	4	-	132.17 (202)	115.56 (160)	61 (46)	2.17 (7)	-	[73]
AZ31	4	90	1500	-	3	-	255 (275)	-	-	-	-	[80]
AZ31	8	120	1200	Conical pin	2	2.5	225.1 (249.5)	130.5 (156.3)	-	5.4 (14)	-	[11]
AZ31-O	2	200	1000	-	2.6	-	~170 (~250)	~90 (~150)	-	-	-	[68]
AZ31B	5	0.5	1000	Tapered cylindrical pin	3	-	183 (262)	101 (179)	-	-	No defects	[74]
AZ31B	5	40	1400	Threaded conical pin	3	2.5	186.76 (215)	139.1 (171)	71 (69)	5.00 (14.7)	-	[81]
AZ31B	5	40	1120	Taper threaded pin	3	2	188 (215)	148 (171)	121 (69.3)	7.3 (14.3)	-	[19]
AZ31B	6	40.2	1600	Threaded cylindrical pin	3.0	0	205 (215)	166 (171)	75 (69.3)	7.3 (14.7)	Defect free	[82]
AZ31B	6	50.8	1200	-	-	-	248	-	67.95 HB	-	-	[71]
AZ31B-O	5	60	1200	Left handed threaded pin	3	-	187.8 (206)	-	64.77 (50)	16.73 (20)	-	[13]
AZ31-H24	3.175	204	2000	-	-	-	225.6 (307.7)	115.3 (227.6)	-	-	No defects	[75]
AZ31B-H24	4.95	4	1000	-	-	-	208 (315)	115 (202)	-	-	-	[83]
AZ61	4	25	1400	Left-handed threaded pin with three flutes	3	-	220 (270)	175 (219)	81 (70)	7.2 (~8.2)	-	[16]
AZ91	6	28	710	Threaded straight cylindrical pin	3	-	76.17	-	-	-	No defects	[84]
AZ91	6	60	600	-	2.8	2.5	262 (106)	132 (55)	-	18.9 (15.2)	No defects on the top surface	[85]
AZ91D	3	75	500	Left-handed tapered cylindrical pin	2.6	2.5	107 (107)	-	-	-	Defect free	[86]
AZ91D	3	90	1200	-	2	-	200 (220)	140 (150)	-	2 (3.6)	-	[87]
MB3	3	120	1500	-	-	-	240 (245)	-	-	-	No macro defects	[88]
Mg-Y-Nd alloy (double side welded)	20	240	700	Threaded conical pin with three flutes	2	3	277.6 (336.1)	204.1 (245.9)	-	7.27 (10.43)	Defect free	[89]

**Table 3:** FSW of steel and ferrous alloys—process parameters and mechanical properties of the joints.

Material	Plate Thickness [mm]	Process Parameters					Weld Properties					Reference
		v [mm/min]	ω [rpm]	Tool Shape	Pin D/d Ratio	Tilt Angle [°]	UTS [MPa]	Yield Strength [MPa]	Hardness of the Stir Zone [HV]	Elongation [%]	Defects	
HSLA-65	6	154.2	600	Convex step-spiral scroll shoulder	-	-	852 (538-690)	662 (448)	-	22.3 (min 18)	-	[102]
HSLA DMR-249A	5	30	600	Tapered pin with no threads	5	0	664 (610)	-	410 (270)	19 (29)	Free from macro-level defects	[108]
IF	1.6	400	400	Cylindrical pin with no threads	3	3	~310 (284)	-	-	-	-	[104]
S12C	1.6	400	400	Cylindrical pin with no threads	3	3	~480 (317)	-	-	-	-	[104]
S35C	1.6	200	400	Cylindrical pin with no threads	3	3	~780 (574)	-	-	-	-	[104]
DH36	6	450	600	-	-	0	832.51 (531.62)	656.68 (376.71)	-	5.57	-	[106]
DH36	6.4	306	526	Slightly tapered pin with no threads	-	2.5	~940 (~580)	~650 (~350)	-	-	No volumetric defects	[90]
Ultrafine grained AISI 304L	2	80	630	Conical pin	~3	3	~760 (920)	~500 (720)	285 (330)	~42 (47)	-	[109]
AISI 316	4	8	1100	-	-	-	610 (608)	-	230 (190)	35 (49)	-	[105]
AISI 316	4	8	1000	-	-	-	630 (608)	-	-	37 (49)	Defect free	[110]
AISI 1018	5	50	1000	Tapered pin with no threads	2.2	-	457 (421)	424 (361)	-	20 (27)	-	[111]
HNAS (High nitrogen nickel-free austenitic stainless steel)	2.4	100	400	Tapered pin	3.3	0	~1100 (~1060)	~760 (~680)	400 (370)	~37 (~43)	No groove-like defects	[112]
Fe-18,4Cr- 15,8Mn-2,1Mo-0,66N-0,04C	2	100	800	-	3	-	980 (967)	580 (604)	-	30 (53)	-	[113]
Fe-18Cr-16Mn-2Mo-0,85N	3	50	800	-	-	2	1375 (1234)	908 (782)	-	25.13 (39.8)	-	[114]
Fe-24Ni-0,1C	1.6	400	200	-	3	3	1283 (793)	390 (336)	-	29.0 (5.6)	-	[103]

**Table 5:** FSW of copper and its alloys—process parameters and mechanical properties of the joints.

Material	Plate Thickness [mm]	Process Parameters					Weld Properties					Reference
		v [mm/min]	ω [rpm]	Tool Shape	Pin D/d Ratio	Tilt Angle [°]	UTS [MPa]	Yield Strength [MPa]	Hardness of the Stir Zone [HV]	Elongation [%]	Defects	
Pure copper	2	50	1200	Conical pin	2	0	217.56 (237.81)	-	-	2.03 (39)	Defect free	[156]
Pure copper	2	30	1000	-	-	-	231 (273)	1.2 (3.1)	136	-	Defect free	[157]
Pure copper	3	30	2000	Tapered pin	2.9	-	~195 (217)	-	-	-	Defect free	[152]
Pure copper	3	100	400	Right hand threaded cylindrical pin and concave shoulder	4	-	282 (282)	-	-	16.4	Defect free	[151]
Pure copper	3	25	1100	-	-	2.5	194 (212)	70 (68)	-	22.8 (28.1)	-	[158]
Pure copper	3	40	900	Flat shoulder and cylindrical pin	3	3	168 (260)	109 (231)	85 (110)	13.5 (31)	Defect free	[159]
Pure copper	3	250	300	Non-threaded cylindrical pin	2.4	-	328 (270)	261 (209)	113.6 (84.6)	23 (22)	Defect free	[150]
Pure copper	4	61	1250	-	-	3	~225 (~260)	-	~90 (105-110)	-	Defect free	[144]
Pure copper	5	30	910	Straight cylindrical pin	3	-	216.9	-	77.37 HB	9.2	No defects	[160]
Pure copper	5	40	580	Taper pin	~3	0	220.7 (261.2)	101.3 (232.0)	-	-	Defect free	[143]
Pure copper	5	75	600	-	-	-	221 (234)	127 (178)	88 (107)	43 (47)	-	[146]
Pure copper	5	50	400	-	-	2.5	235.9 (236.7)	207.7 (222.9)	-	15.1 (27.7)	Defect free	[145]
Pure copper	5	50	800	Cylindrical threaded pin	~3.3	2.5	~240 (~240)	~140 (~225)	63.1 (82.2)	~45 (~28)	Defect free	[153]
Pure copper	5	112	500	Threaded cylindrical pin	2.5	2.5	326 (331)	134 (137)	~105 (~78)	31 (29)	Defect free	[161]
Pure copper	6	50	350	Threaded cylindrical pin	3	0	228 (279)	209 (271)	92 (82)	-	-	[155]
Pure copper	6	50	350	Square pin	-	0	207 (279)	203 (271)	88 (82)	-	-	[155]
Pure copper	6	50	350	Triflute pin	-	0	196 (279)	196 (271)	90 (82)	-	-	[155]
Pure copper	6	50	350	Hexagonal pin	-	0	165 (279)	163 (271)	97 (82)	-	-	[155]
Pure copper	6	50	500	Concave shoulder and threaded cylindrical pin	3	0	229 (279)	229 (271)	88 (82)	49.9 (34.4)	Defect free	[154]
Pure copper	6	315	630	Square pin	2.4	2	215 (150)	190 (145)	-	33 (14)	-	[162]
2200 Copper Alloy	5	31.25	900	Taper pin with no threads	3.3	1	-	-	-	26.98	-	[163]
2200 Copper Alloy	5	31.25	900	Cylindrical with no threads	3.3	1	-	-	-	10.56	-	[163]
Brass 60%-Cu, 40%-Zn	2	500	1000	-	3	3	~390 (381)	~180 (192)	~132 (97)	~52 (61)	Defect free	[141]

**Table 6:** FSW of polymers—process parameters and mechanical properties of the joints.

Material	Plate Thickness [mm]	Process Parameters					Weld Properties					Reference
		v [mm/min]	ω [rpm]	Tool Shape	Pin D/d Ratio	Tilt Angle [°]	UTS [MPa]	Yield Strength [MPa]	Hardness of the Stir Zone [HV]	Elongation [%]	Defects	
Polyethylene (PE)	5	25	1000	Cylindrical pin with no threads	3.2	1	19.30 (20.00)	-	-	-	-	[165]
Polypropylene (PP)	6	15	750	Square pin with no threads	2	1	19.74 (33)	-	-	-	Peeling defects	[180]
Polyvinyl chloride (PVC)	5	10	1800	Right-hand screw pin	~4.2	0	23.5 (66.5)	-	Around 78% of base material	-	-	[171]
Nylon 6	10	10	1000	Left handed threaded cylindrical pin	4	-	34.8 (73.4)	-	64 SD (shore-D hardness) (70 SD)	-	Defect free	[173]
Nylon 6	13	10	1250	Right handed threaded cylindrical pin	-	-	25.75 (54)	-	-	8.7 (43)	-	[175]
Nylon 6	16	25	300	Right-hand threaded pin	2.4	0	27.22 (85)	-	-	-	Lack of bonding, minor weld defects at the bottom	[174]
Polyamide 6 (PA6)	5	40	440	Right-hand screw pin	~4.2	0	30 (67.1)	-	60% of base material	-	-	[171]
Polyamide (nylon 66)	8	42	1570	Smooth cylindrical pin	4	-	8.51 (15.57)	-	-	-	-	[193]
Acrylonitrile Butadiene Styrene (ABS)	5	20	1600	Right-hand threaded cylindrical pin	1.7	-	32.62 (36.76)	-	-	-	-	[176]
Acrylonitrile Butadiene Styrene (ABS) (double side welded)	5	40	400	Smooth cylindrical pin, flat shoulders surfaces	-	-	15.58 (34.14)	-	-	-	-	[178]
Acrylonitrile Butadiene Styrene (ABS) (double side welded)	5	40	400	Smooth convex pin, flat shoulders surfaces	-	-	20.70 (34.14)	-	-	-	-	[178]
Acrylonitrile Butadiene Styrene (ABS)	6	200	1500	Conical threaded pin	-	-	30.6 (40.5)	-	-	-	Defect free	[179]
Acrylonitrile Butadiene Styrene (ABS)	8	16	1400	Cylindrical with no threads	3.3	1	41.42 (41.80)	-	-	-	-	[177]
Acrylonitrile Butadiene Styrene (ABS)	8	25	900	Conical with no threads	3.3	2	41.95 (41.80)	-	-	-	-	[177]
High-density polyethylene (HDPE)	4	115	3000	-	3	2	19.4 (22.5)	-	-	-	-	[166]
High-density polyethylene (HDPE)	5	15	1240	Right-hand screw pin	~4.2	0	22.3 (31.9)	-	Above 90% of base material	-	No significant defects	[171]

**Table 7:** FSW of composites—process parameters and mechanical properties of the joints.

Material	Plate Thickness [mm]	Process Parameters					Weld Properties					Reference
		v [mm/min]	ω [rpm]	Tool Shape	Pin D/d Ratio	Tilt Angle [°]	UTS [MPa]	Yield Strength [MPa]	Hardness of the Stir Zone [HV]	Elongation [%]	Defects	
Al-4.5%Cu/TiB2/2.5p	6	26	931	Full flat shoulder surface, cylindrical pin with no threads	~2.9	2	190.39 (no inf about parent material)	-	69.86	16.875	-	[213]
Al-4.5%Cu/TiB2/2.5p	6	26	931	1 mm flat shoulder and 7° concave, cylindrical pin with no threads	~2.9	2	198.62	-	57.63	18.825	-	[213]
Al-4.5%Cu/TiB2/2.5p	6	26	931	2 mm flat shoulder and 7° concave, cylindrical pin with no threads	~2.9	2	191.39	-	60.76	17.050	-	[213]
AA6061/SiC (10wt%) /fly ash (7.5 wt%)	6	60	1200	Square profile pin	-	2	-	-	~130 (102)	-	No major defects	[214]
AA2124/SiC/25p-T4	3	40	1120	Cylindrical left-handed screwed pin	3.3	2	366 (454)	-	170 (185)	1.4 (2.4)	-	[215]
AA6092/SiC/17.5p	6	120	1500	Taper cylindrical pin	3	2	347 (415)	290 (360)	140 (157)	5.46 (7.76)	No major defects	[208,216]
AC4A + 30vol%SiCp	5	150	2000	Columnar pin with right-handed threads	2.3	3	140 (163)	-	-	0.33	Defect free	[211]
17vol%SiCp/2009Al-T4	3	800	1000	Threaded conical pin	2.8	-	501 (514)	341 (344)	-	3.5 (4.0)	No defects	[212]
17vol%SiCp/AA2009	3	50	1000	Cylindrical pin	2.8	-	443 (581)	278 (508)	-	4.7 (4.3)	No defects	[217]
AA2124/SiC/25p-T4	3	45	900	-	3	2	355.15 (454)	-	-	-	Flash defects on the surface	[218]
25%SiC/2124Al	8	15	400	Tapered conical pin	3.5	1.5	359 (372)	-	-	-	Small voids	[219]
AA6061-10%SiCp	6	45	1100	Threaded cylindrical pin	3	-	206 (278)	126 (200)	95 (105)	6.5 (8.0)	No defects	[220]
15vol%SiCp/2009Al	6	100	800	Conical pin	2.5	-	441 (537)	306 (343)	-	5.4 (10.1)	No defects	[221]
AA6092/SiC/17.5p-T6	3.1	100	1500	Flat edge featureless concave shoulder and M6 threaded cylindrical pin with a domed end	-	2	314 (420)	220 (370)	-	5 (3.5)	-	[222]
AA6082/SiC/10p	6	100	1800	Cylindrical	2.5	1	359	-	-	-	-	[209]
LM25AA-5% SiCp	12	40	1000	Plain taper pin	3	-	192 (155)	-	~105 (68)	7.2 (2)	-	[116]
PP/C30B/EA nanocomposite	5	18	-	Square pin with no threads	1	-	13.533 (25.08)	-	-	-	-	[210]
PP/C30B/EA nanocomposite	5	18	-	Cylindrical pin with no threads	1	-	16.300 (25.08)	-	-	-	-	[210]
PP/C30B/EA nanocomposite	5	18	-	Triangle pin with no threads	1	-	13.500 (25.08)	-	-	-	-	[210]
Glass-filled Nylon 6	5	12	600	Cylindrical pin with cylindrical shank	3	2	36.51 (86.01)	-	-	7.35 (13.68)	Defect free	[208]

**Table 8:** FSW of dissimilar materials—process parameters and mechanical properties of the joints.

Material		Plate Thickness [mm]	Process Parameters					Weld Properties					Reference
Advancing Side	Retreating Side		v [mm/min]	ω [rpm]	Tool Shape	Pin D/d Ratio	Tilt Angle [°]	UTS [MPa]	Yield Strength [MPa]	Hardness of the Stir Zone [HV]	Elongation [%]	Defects	
AA2024-T3	AA7075-T6	3	102	1200	Cylindrical threaded pin	3	-	423 (416 for AA2024-T3 and 593 for AA7075-T6)	290.0 (327 for AA2024-T3 and 498 for AA7075-T6)	-	14.9 (29.5 for AA2024-T3 and 17.7 for AA7075-T6)	-	[244]
AA2024-T6	AA7075-T6	5	12	1200	Flat shoulder and smooth cylindrical pin	3	-	356 (416 for AA2024-T6 and 485 for AA7075-T6)	-	-	-	Defect free	[116]
AA5052	AA6061	-	28	710	Cylindrical pin with two threads	3	-	180	-	82	10.6	-	[248]
AA5083	AA6082	3	300	840	Conical surface of the shoulder, threaded cylindrical pin	3	2	241	-	-	3.05	-	[243]
AA5086-O	AA6061-T6	5	150	840	Tapered pin with 3 threads and concave shoulder surface	3.3	-	221 (253 for AA5086-O; 315 for AA6061-T6)	136 (112 for AA5086-O; 278 for AA6061-T6)	-	-	-	[31]
AA6061-T6	AA5086-O	5	150	840	Tapered pin with 3 threads and concave shoulder surface	3.3	-	224 (315 for AA6061-T6; 253 for AA5086-O)	139 (278 for AA6061-T6; 112 for AA5086-O)	-	-	-	[31]
AA6082	AA5083	3	200	840	Conical surface of the shoulder, threaded cylindrical pin	3	2	227	-	-	2.67	-	[243]
AA6351-T6	AA5083-H111	6	60	950	Flat shoulder surface, straight square pin with no threads	3	0	273 (AA6351-T6: 310 AA5083H111: 308)	-	-	-	No defects	[246]
AA7075-T6	AA2024-T3	6.5 (AA7075-T6); 5 (AA2024-T3)	80	1400	Square pin	-	-	261	-	-	-	Defect-free	[245]
AA7075-T6	AA2024-T3	3	102	1200	Cylindrical threaded pin	3	-	381 (593 for AA7075-T6 and 416 for AA2024-T3)	280.0 (498 for AA7075-T6 and 327 for AA2024-T3)	-	9.0 (17.7 for AA7075-T6 and 29.5 for AA2024-T3)	-	[244]
St37	304 austenitic stainless steel	3	50	600	-	2.9	3	494 (446 for St37 and 679 for 304 steel)	290 (305 for St37 and 287 for 304 steel)	240 (120 for St37 and 180 for 304 steel)	28 (42 for St37 and 111 for 304 steel)	No macro defects	[249]
St52 mild steel	AA5186	3	56	355	M3 threaded pin	6	3	246 (520 for St52 and 275 for AA5186)	-	-	-	No defects	[250]
Low carbon steel	Pure Mg	2	100	1000	Cylindrical with no threads	3	3	~70 (316 for low carbon steel and 170 for pure Mg)	-	-	-	-	[242]
Low carbon steel	AZ31	2	100	500	Cylindrical with no threads	3	3	~165 (316 for low carbo steel and 260 for AZ31)	-	-	-	-	[242]
Low carbon steel	AZ61	2	100	750	Cylindrical with no threads	3	3	~220 (316 for low carbon steel and 280 for AZ61)	-	-	-	-	[242]
AZ31	AA6040-T61	2	225	1400	Threaded tapered pin	2.6	2.5	189 (228–238 for AZ31 and 175-205 for Al6040-T61)	-	-	-	-	[241]
AA6040-T61	AZ31	2	200	1400	Threaded tapered pin	2.6	2.5	127 (175–205 for Al6040-T61 and 228–238 for AZ31)	-	-	-	-	[241]
AA5052-H	AZ31B	3	200	1000	-	3	3	147 (244 for A5052-H and 241 for AZ31B)	64.0 (181 for A5052-H and 200 for AZ31B)	-	3.4 (18.0 for A5052-H and 21.6 for AZ31B)	No defects	[251]
AA6061	AZ31	3	40	1000	Concave shoulder and cylindrical pin	5	2.5	178 (295 for 6061 Al and 235 for AZ31)	170 (235 for 6061 Al and 130 for AZ31)	-	2.4 (12.5 for 6061 Al and 18.7 for AZ31)	Defect free	[252]
AZ31B-0	AA6061-T6	6	20	400	Tapered smooth pin	3.5	-	192 (216 for AZ31B-0 and 311 for AA6061-T6)	153 (175 for AZ31B-0 and 280 for AA6061-T6)	-	10 (15 for AZ31B-0 and 20 for AA6061-T6)	Defect free	[247]
AZ31B	AA1100	3	20	570	Cylindrical threaded pin	3.6	-	122 (228 for AZ31B; 175 AA1100)	101 (145 AZ31B; 105 AA1100)	-	9 (17 AZ31B; 11 AA1100)	-	[253]
SS400 mild steel	AZ31B-O	2	100	1250	Unthreaded cylindrical pin	5	-	178.5 (455 for SS400; 257 AZ31B-O)	-	-	~2.7 (39 for SS400 and 23.3 for AZ31B-O)	-	[254]
304L stainless steel	Pure Cu	3	31.5	1500	Taper pin	-	2	173.05 (574 for 304L; 227 for copper)	-	-	8.22 (51 for 304L; 23 for copper)	-	[255]
Pure Cu	AA1050	6	63	1400	-	-	-	88.466 (91% of AA1050)	-	-	-	-	[256]
Pure Cu	AA1060	5	100	600	Cylindrical pin	~3.3	-	110	-	-	-	Defect free	[257]
